# Supplementary material for: Platinum Chemotherapy Induces Lymphangiogenesis in Cancerous and Healthy Tissues That Can be Prevented With Adjuvant Anti-VEGFR3 Therapy
Source: Front Oncol. 2022 Mar 17;12:801764. doi: 10.3389/fonc.2022.801764 (PMC8970967; doi:10.3389/fonc.2022.801764)
Supplement: Supplementary file 1 [file DataSheet_1.pdf]

Figure S1 | Lymphatic endothelial cell morphology in response to chemotherapies

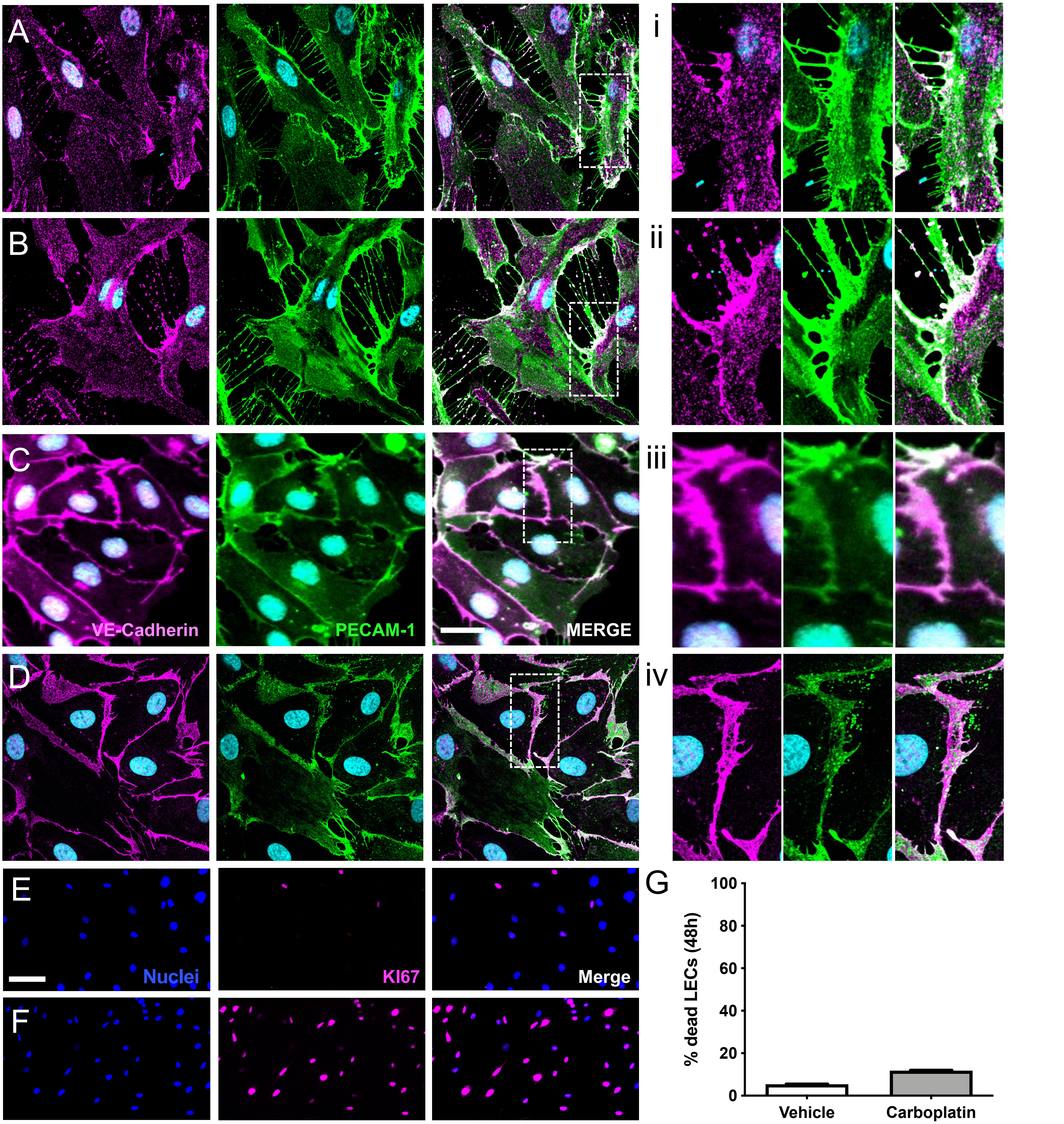

Supplemental Figure 1 | Lymphatic endothelial cell morphology in response to chemotherapies. (A-D, i-iv) Representative images of LEC morphology and junctions after platinum chemotherapy. Human LECs were treated *in vitro* with 1 µM of **(A)** cisplatin, **(B)** oxaliplatin, **(C)** doxorubicin, or **(D)** docetaxel for 6 hours. Cell morphology and junctions were visualized by immunocytochemical staining of VE-cadherin (magenta) and PECAM-1/CD31 (green) with DAPI (cyan) for nuclei. Junctions were imaged by high-powered confocal microscopy (scale bar = 25 microns). **(i-iv)** High magnification images of boxed areas in A-D. **(E-F)** Representative images of human LECs treated *in vitro* with **(E)** vehicle or **(F)** 1 µM of carboplatin and assessed for proliferation after 6 hours by immunocytochemical staining of KI67 (pink). (**G**) LEC viability after 48 hours of continuous treatment. Data represented as percentage of dead LECs as assessed by staining with live/dead viability dye, n=2.

Figure S2 | Blood vasculature in a mesentery explant model

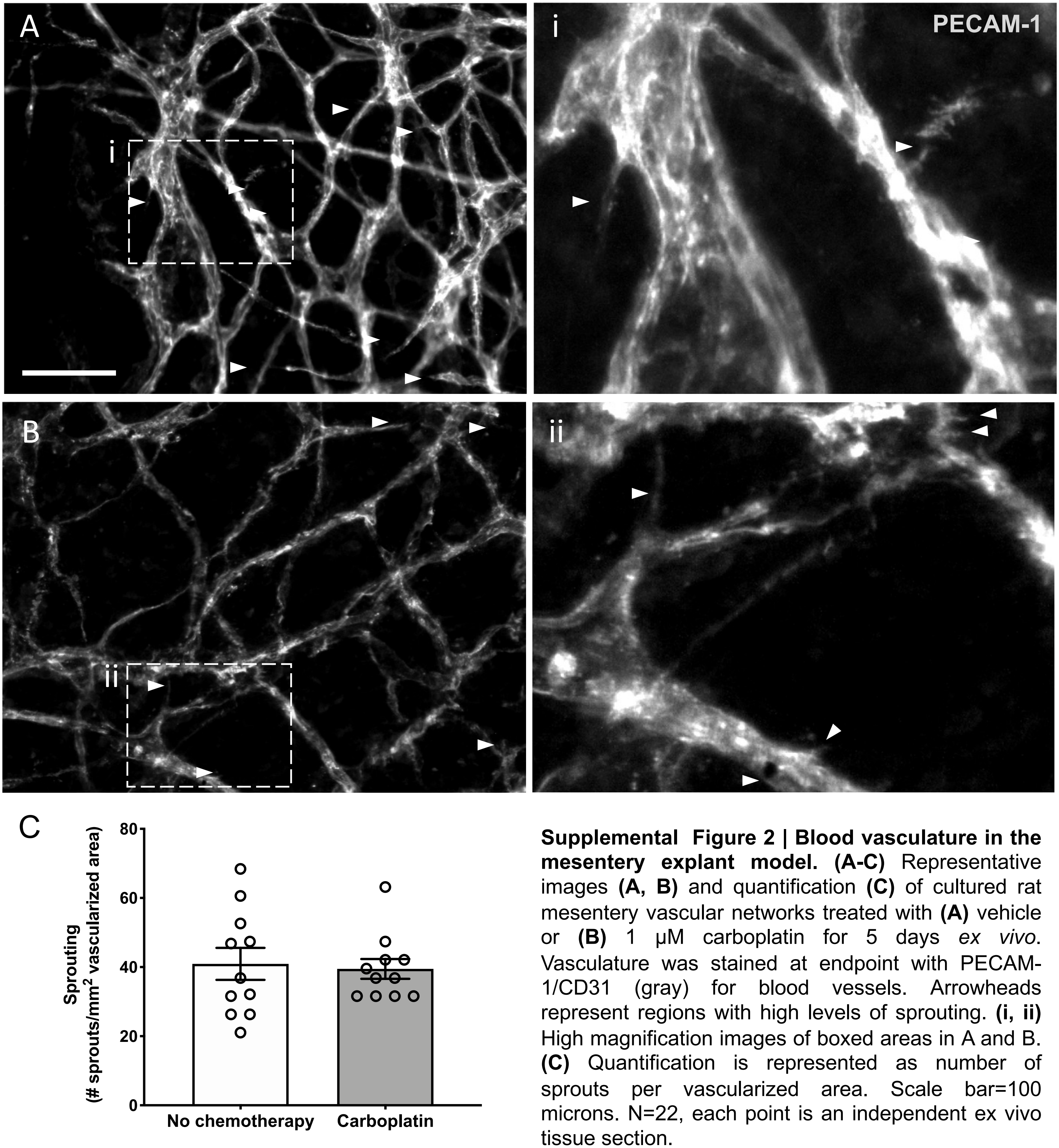

Supplemental Figure 2 | Blood vasculature in the mesentery explant model. (A-C) Representative images **(A, B)** and quantification **(C)** of cultured rat mesentery vascular networks treated with **(A)** vehicle or **(B)** 1 μM carboplatin for 5 days *ex vivo*. Vasculature was stained at endpoint with PECAM-1/CD31 (gray) for blood vessels. Arrowheads represent regions with high levels of sprouting. **(i, ii)** High magnification images of boxed areas in A and B. **(C)** Quantification is represented as number of sprouts per vascularized area. Scale bar=100 microns. N=22, each point is an independent ex vivo tissue section.

Figure S3 | Multiple parameters of lymphatic vessels can be measured and quantified using specialized Matlab tool

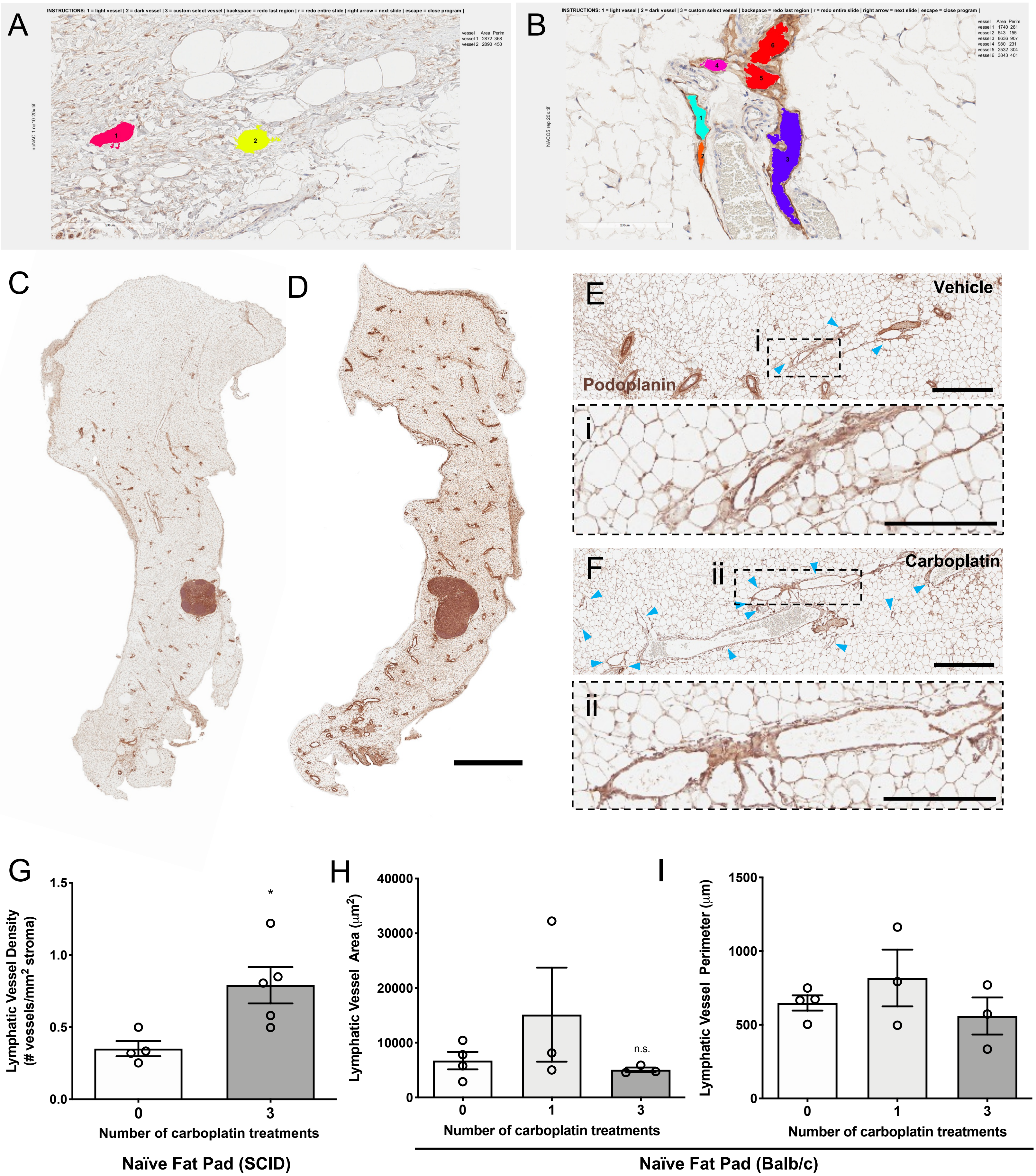

Supplemental Figure 3 | Multiple parameters of lymphatic vessels can be measured and quantified using specialized Matlab tool. (A, B) Representative images of lymphatic vessel quantification program using MathWorks Matlab tool. Colored and numbered regions are lymphatic vessels quantified by the software program. Vessel area and perimeter output can be seen on the far right of the window. **(A)** represents a sample with no chemotherapy while **(B)** represents a sample with chemotherapy. **(C-F, i-ii)** Representative images of mammary fat pads from naïve mice treated with 0, 1, or 3 doses of **(C)** vehicle or **(D)** 8 mg/kg systemic carboplatin by IV. Naïve fat pads were harvested three days following the final treatment and stained for lymphatic vessel marker podoplanin by immunohistochemistry. Representative images of lymphatic vessels are shown in **E** (vehicle) and **F** (carboplatin), with blue arrowheads showing lymphatic vessels (scale bar = 300 microns). (**i, ii**) High magnification images of lymphatic vessels from boxed regions (scale bar = 200 microns). **(G)** Immunocompromised NOD/SCID mice were injected with three doses of carboplatin (8 mg/kg, IV) or vehicle. Naïve mammary fat pads were harvested one week following final treatment and assessed for lymphatic vessel density by immunohistochemistry as previously described. Quantification of LVD in naïve mammary fat pad represented by total number of lymphatic vessels per stromal area. *$p<0.05$ as analyzed by individual *t* tests, N=9 mice total. **(H, I)** Quantification of lymphatic vessel (**H**) area and (**I**) perimeter in naïve balb/c MFPs treated as described in C-F from experiment shown in Figure 2E. Each data point represents one mouse.

Figure S4 | Lymphangiogenesis with carboplatin treatment in naïve tissues

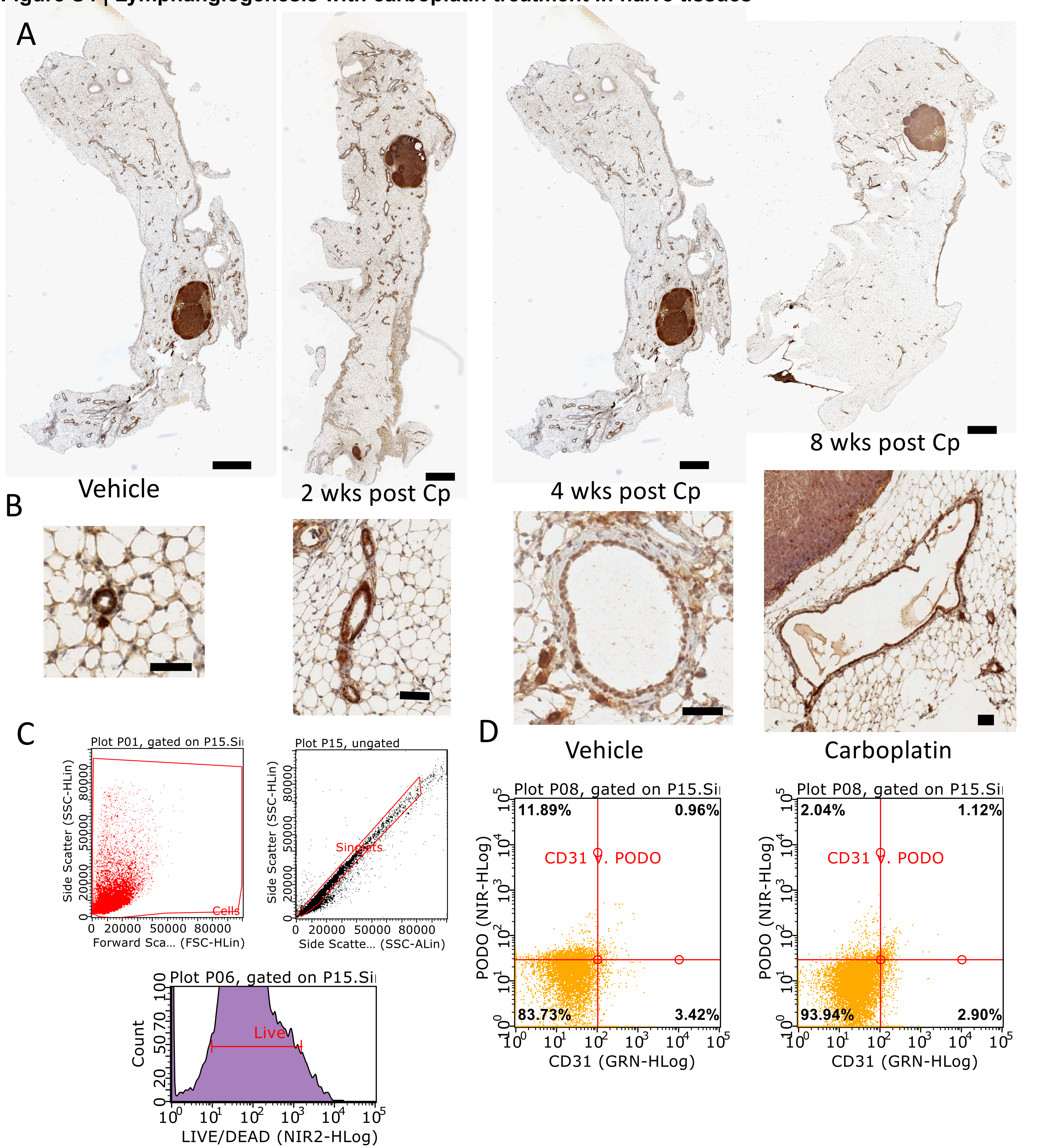

Supplemental Figure 4 | Lymphangiogenesis with carboplatin treatment in naïve tissues. (A) Representative images of mammary fat pads from naïve Balb/c mice treated with 3 doses of vehicle or 8 mg/kg systemic carboplatin by IV. Naïve fat pads were harvested two, four or eight weeks following the final treatment and stained for lymphatic vessel marker podoplanin by immunohistochemistry (scale bar=1 mm). **(B)** Representative images of lymphatic vessels at the same timepoints (scale bar = 50 microns). **(C)** Representative gating scheme for draining lymph nodes after three injections of 5 mg/kg carboplatin or vehicle into the inguinal mammary fat pad. Cells were gated for total cells, singlets, and live cells first. **(D)** LEC populations were identified by co-staining for CD31 and podoplanin shown here for one representative sample for each treatment. Graphed data with experimental information is shown in Figure 2F,H.

Figure S5 | Lymphatic analysis of 4T1 and xenograft mammary fat pads

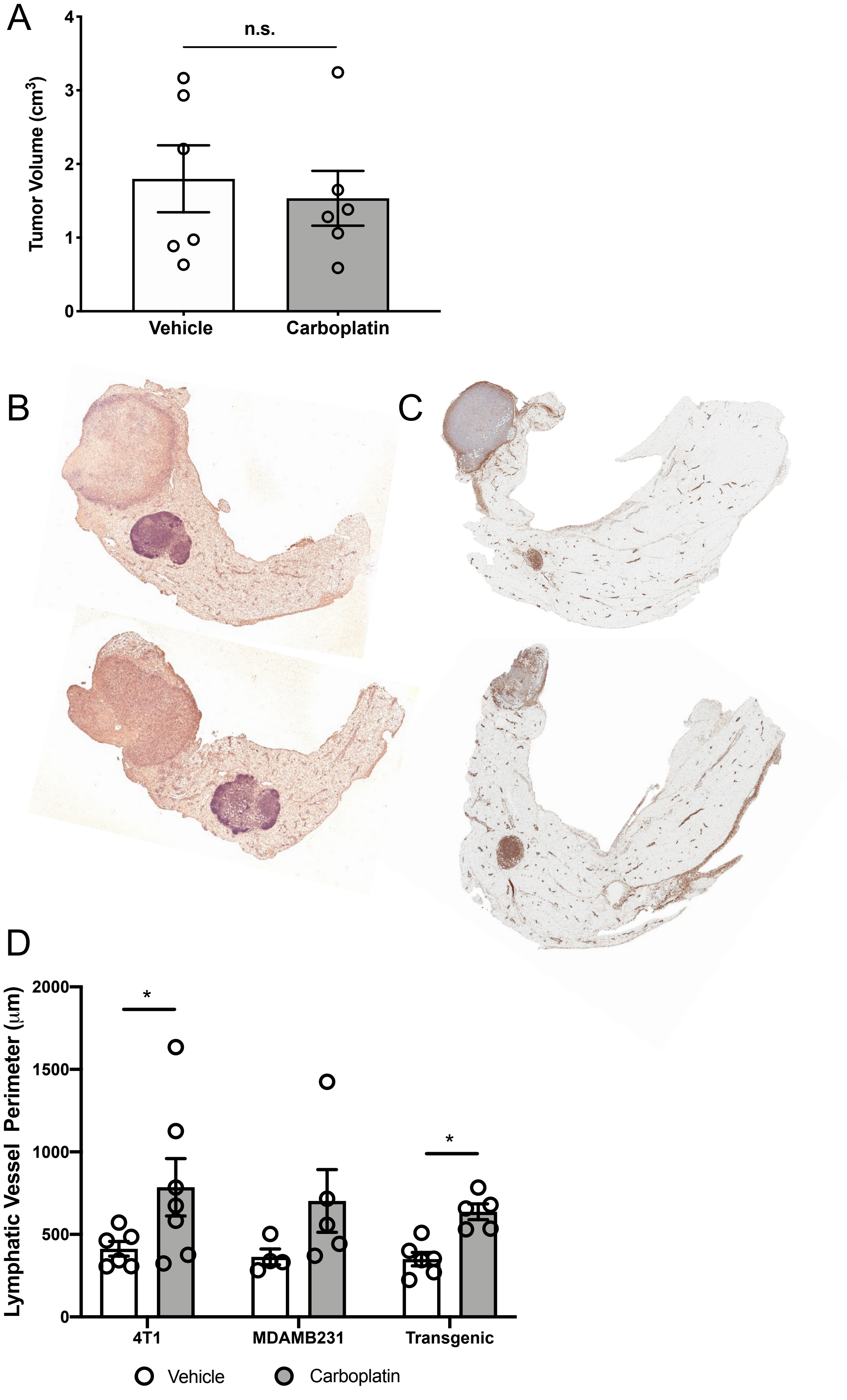

Supplemental Figure 5 | Lymphatic analysis of 4T1 and xenograft mammary fat pads (A-D) In all studies, mice were treated with carboplatin (8 mg/kg, 3 doses, IV) when tumors were palpable; mice were euthanized once the largest tumor within each study reached predetermined size endpoint. **(A)** Representative tumor weights at endpoint from autochthonous mammary tumor model, L-Stop-K-*KRas*^{G12D}*p53*$^{flx/flx}$-L-Stop-L-*Myristoylated p110α*-GFP^{+} (induced by intraductal injection of adenovirus-Cre) show no difference in final tumor weight after treatment due to the low dose of carboplatin used. Representative tumor-bearing MFPs from **(B)** 4T1 (top, vehicle; bottom, carboplatin) and **(C)** xenografted MDAMB231 (top, vehicle; bottom, carboplatin) mouse models. **(D)** Quantification of lymphatic vessel perimeter from all tumor models treated as described in Fig. 1 and S1. *$p<0.05$ as analyzed by two-way ANOVA, n=4-6 mice/cohort.

Figure S6 | Priming of tissue with carboplatin prior to tumor inoculation increases lung metastasis without altering tumor growth.

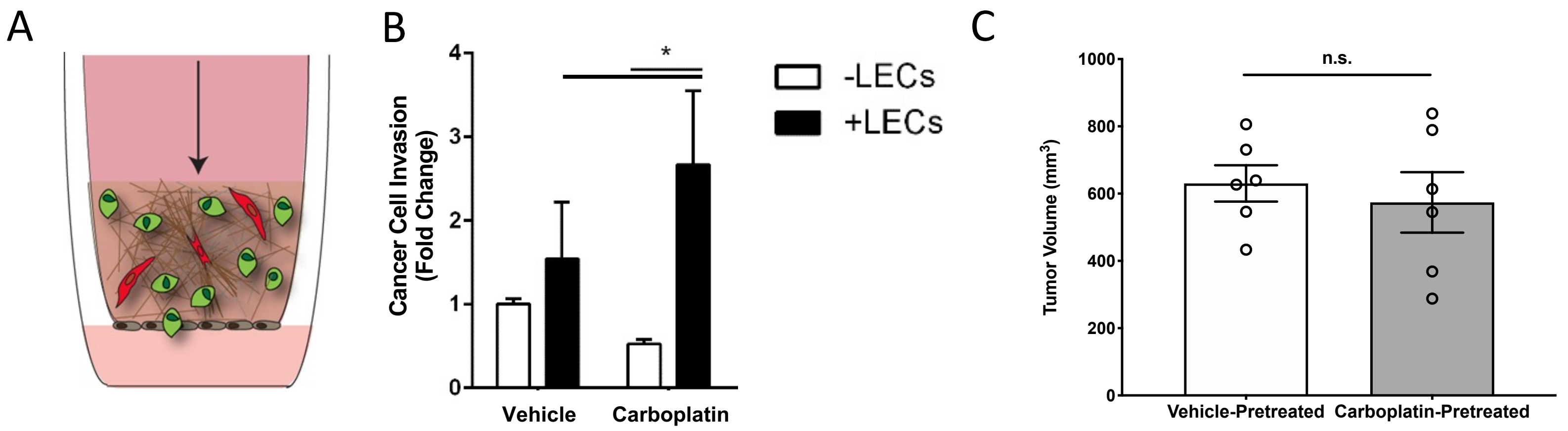

Supplemental Figure 6 | Priming of tissue with carboplatin prior to tumor inoculation increases lung metastasis without altering tumor growth. (A) Schematic of the *in vitro* tissue engineered model of the human breast cancer microenvironment including human triple-negative breast tumor cells, human lymphatic endothelial cells (LECs), and human mammary fibroblasts in a 3D collagen I matrix atop a porous tissue culture insert. Physiologically-relevant fluid flow with or without carboplatin is applied through the system via a pressure head. Schematic depicts experimental groups. **(B)** Fold change in invasion of MDA-MB-231 tumor cells across the porous membrane in our 3D microenvironment system +/- carboplatin treatment (1 μM) and/or in the presence or absence of LECs. *$p<0.05$, n=4 independent experimental runs. **(C)** Naïve mice were treated with carboplatin (8 mg/kg, 3 doses, IV) and drug was allowed to clear for one week after final treatment so as not to affect tumor initiation. Mice were then orthotopically implanted with 10,000 4T1 cells by subareolar injection. Tumors were allowed to grow until humane size endpoints were reached; there were no observed differences in final tumor size between cohorts (n=6/group).

Figure S7 | Inhibition of VEGFR3 normalizes LECs *in vitro*

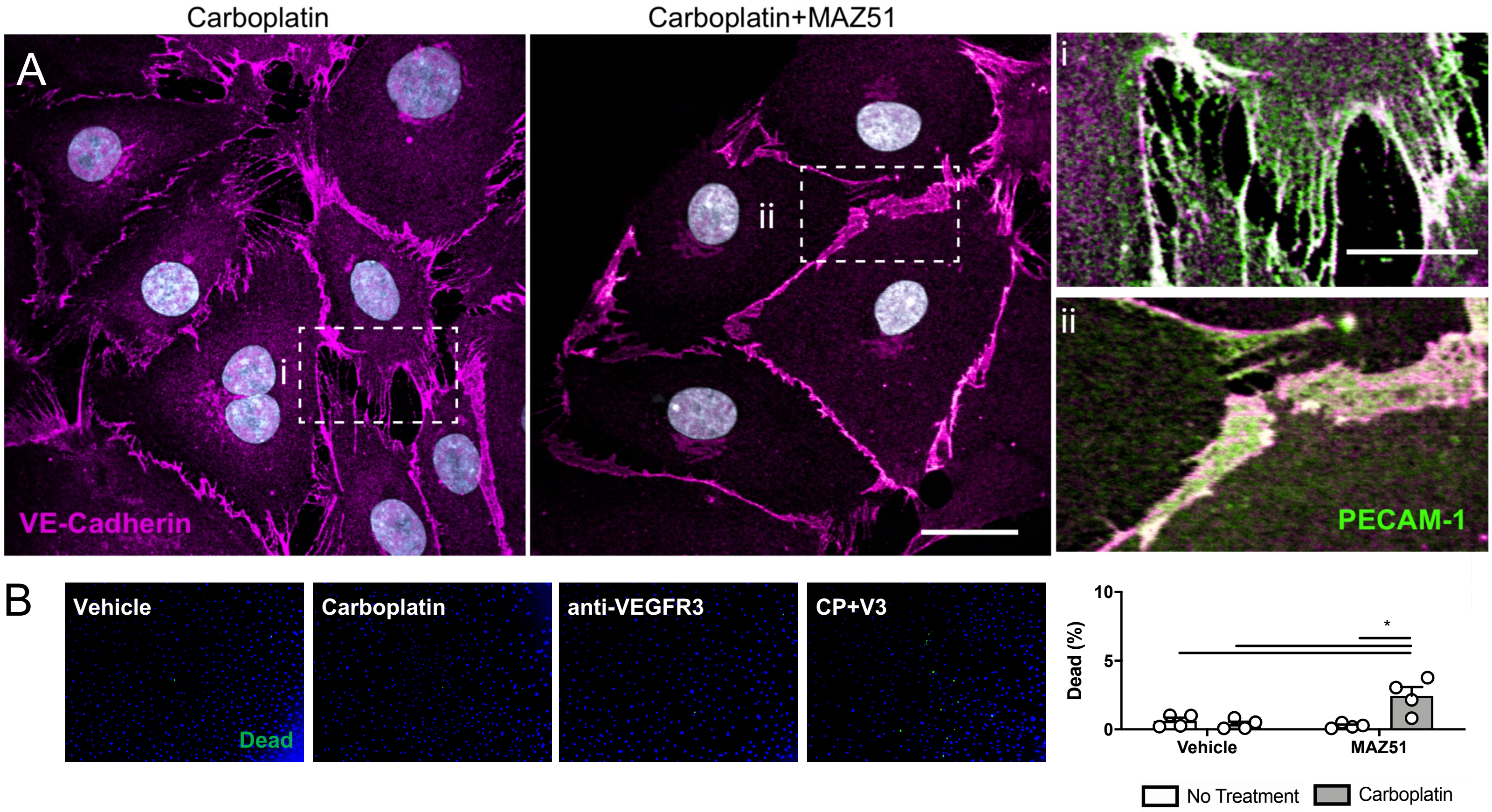

Figure S7 | Inhibition of VEGFR3 normalizes LECs *in vitro*. (A) LEC monolayers after 6h treatment VE-Cadherin (magenta) and PECAM1 (green) with high magnification images of boxed areas (i, ii). **(B)** Representative images and quantification of cell death as measured by percentage of cells dead per field after 48 hours of platinum agent (1μM) and/or MAZ51 and assessed by amine-based fluorescent reactive dye for dead cells. n≧3 per group, *p<0.05

Figure S8 | Inhibition of VEGFR3 mitigates all platinum-induced lymphatic changes

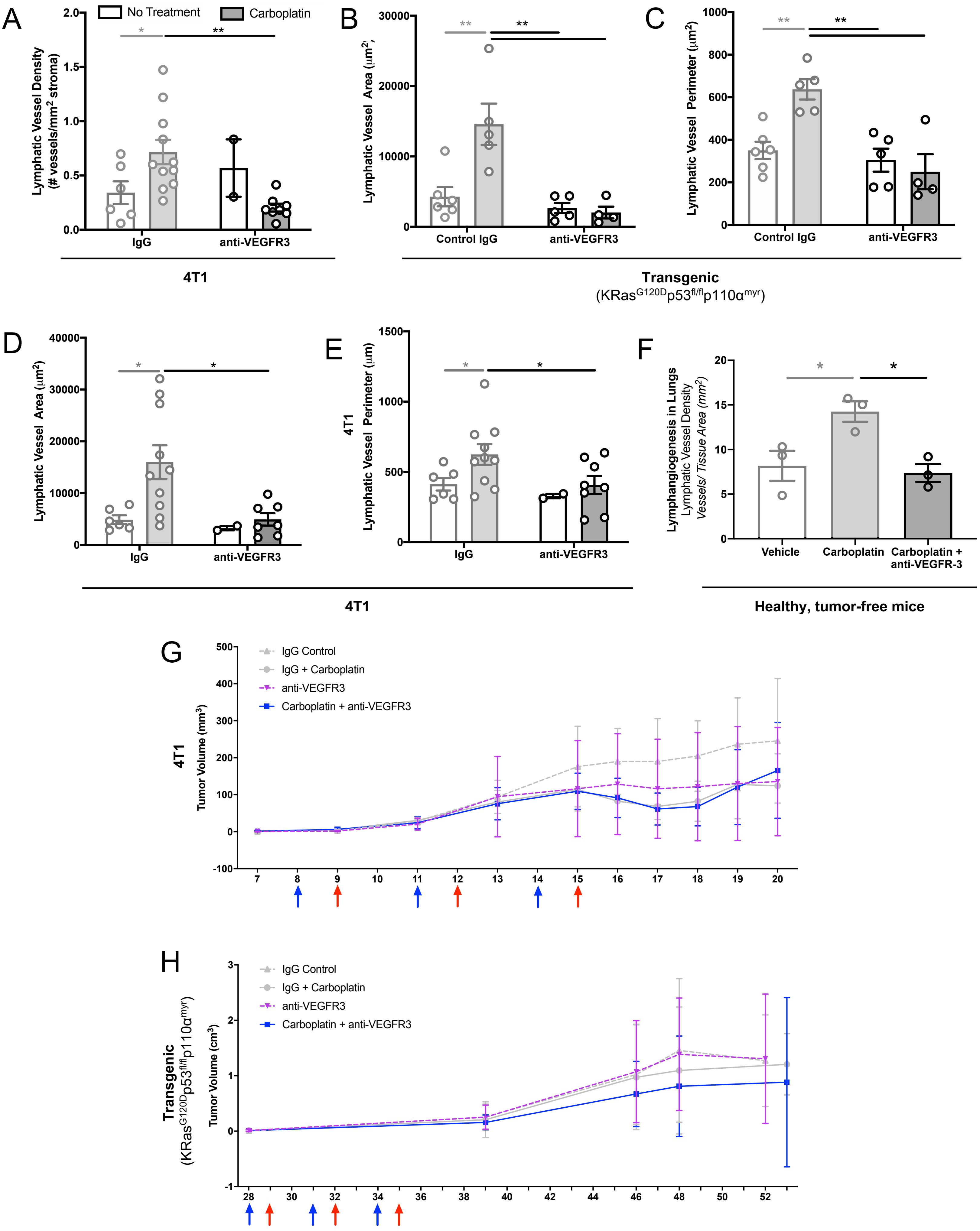

Supplemental Figure 8 | Inhibition of VEGFR3 mitigates all platinum-induced lymphatic changes. (A) Lymphatic vessel density as measured by number of podoplanin+ vessels per mm^2 stroma by immunohistochemical staining of tumor-bearing mammary fat pads from 4T1 mice treated with anti-VEGFR/control IgG antibodies + carboplatin/vehicle as previously described**.** **(B)** Lymphatic vessel area and (**C**) lymphatic vessel perimeter in tumor-bearing mammary fat pads of transgenic $KRas^{G120D}p53^{fl/fl}p110\alpha^{myr}$ mice treated with anti-VEGFR3/control IgG antibodies + carboplatin/vehicle as previously described. **(D)** Lymphatic vessel area and (**E**) lymphatic vessel perimeter in tumor-bearing mammary fat pads of 4T1 mice treated with anti-VEGFR3/control IgG antibodies + carboplatin/vehicle as previously described. **(F)** Naïve, healthy Prox1-tdTomato reporter mice were treated with carboplatin (8 mg/kg, 3 doses, IV) and lungs were collected 3 days following the final treatment. Lungs were fixed, sectioned, and assessed for lymphangiogenesis using image thresholding in Fiji. *$p<0.05$. **(G)** Tumor growth curve for 4T1 mice treated with anti-VEGFR3/control IgG antibodies + carboplatin/vehicle as previously described beginning once tumors were palpable. Blue arrows note antibody treatments, while red arrows note chemotherapy treatments. **(H)** Tumor growth curve for transgenic $KRas^{G120D}p53^{fl/fl}p110\alpha^{myr}$ mice treated with anti-VEGFR3/control IgG antibodies + carboplatin/vehicle as previously described beginning once tumors were palpable. Blue arrows note antibody treatments, while red arrows note chemotherapy treatments. Further information can be found in Figure 6.

Table S1 | Gene enrichment analysis of pathways upregulated in LECs after carboplatin treatment

Gene Enrichment Analysis by STRING	
**Pathways upregulated by carboplatin**	
KEGG Pathway	*Adjusted P-value*
MAPK signaling	3.8E-05
JAK-STAT signaling	4.5E-05
Pathways in cancer	8.9E-05
GAP junctions	1.1E-04
Cell adhesion molecules	4.9E-04
PI3K-AKT signaling	1.0E-03
HIF-1 signaling	1.4E-03
Ras signaling	1.4E-03
Chemokine signaling	1.4E-03

Table S3 | Intersecting pathways of microRNA regulation upregulated in LECs after carboplatin treatment.

MicroRNA Pathway Analysis by DIANA mirPath		
**Intersecting pathways of miRNAs upregulated by carboplatin**		
KEGG Pathway	*P-value*	*% of upregulated miRNAs involved*
TGFb signaling	3.46E-08	33.3%
Adherens junctions	6.21E-08	66.7%
Cell cycle	0.006	44.4%
Proteoglycans in cancer	0.014	66.7%
HIF-1 signaling	0.019	44.4%
**Top miRNAs upregulated by carboplatin (FC>2)**		
MIR3147, MIR130B, MIR3127, MIR4526, MIR4310, MIR421, MIR3116-1, MIR1224, MIR4659A		

Table S2 | Pathways upregulated in LECs after carboplatin by RPPA analysis

RPPA Aanlysis	
**Pathways upregulated by carboplatin**	
Signaling Pathway	*Adjusted P-value*
Mammary gland development	3.36E-05
EGF/EGFR signaling	6.96E-05
Pac1-Pak1-p38-MMP2	7.46E-05
FGF/ FGFR signaling	8.39E-05
ErbB2/ErbB3 signaling	0.000379
Pathways in cancer	0.000651
IL6	0.000995
Pathways in TGF-B signaling	0.00107
HIF-1 signaling	0.0019
Alpha9 beta1 integrin	0.00261
Oxidative stress-induced senescence	0.00292
MicroRNAs in cancer	0.00292
Adherens junctions	0.00329
N-cadherin signaling events	0.00402
A6b1 and a6b4 integrin signaling	0.00546
Regulation of microtubule cytoskeleton	0.00562
Angiopoietin receptor Tie2-mediate signaling	0.00596
Posttranslational regulation of adherens junction stability and dissassembly	0.00615
Focal adhesion	0.00615
Cell-cell junction organization	0.00748
Beta1 integrin cell surface interactions	0.00767
Wnt signaling	0.00797
Alpha6 beta4 integrin	0.00815
DNA double strand break response	0.00871
MAPK signaling pathway	0.012
Cell cycle	0.0137
JAK/STAT signaling	0.0138

Table S4 | Patient cohort characteristics for histologically benign/normal omentum from patients treated with and without platinum chemotherapy.

Patient Characteristics (Benign Tissues)				
Histologically Normal Omentum				
Patient ID	Tissue	Pathological Diagnosis	Neoadjuvant Chemotherapy	LVD (# LV/mm^2 tissue)
O1	Omentum	Negative	None	0.063
O2	Omentum	Negative	None	0.560
O4	Omentum	Negative	None	0.251
NACO0	Omentum	Negative	Carboplatin + Paclitaxel	2.266
NACO1	Omentum	Negative	Carboplatin + Paclitaxel	1.318
NACO2	Omentum	Negative	Carboplatin + Paclitaxel	0.685
NACO3	Omentum	Negative	Carboplatin + Paclitaxel	7.211
NACO5	Omentum	Negative	Carboplatin + Paclitaxel	2.503

Table S5 | Patient cohort characteristics for metastatic omentum from ovarian cancer patients treated with and without neoadjuvant platinum chemotherapy

Patient Characteristics (Tumor Tissues)					
Ovarian Cancer					
Patient ID	Clinical Staging	Tissue	Pathological Diagnosis	Neoadjuvant Chemotherapy	LVD (# LV/mm^2 tissue)
T0	Stage 3	Omentum	Positive	None	0.089
T3	Stage 3	Omentum	Positive	None	0.807
T4	Stage 3	Omentum	Positive	None	0.186
T5	Stage 3	Omentum	Positive	None	0.119
T6	Stage 3	Omentum	Positive	None	0.419
T8	Stage 4	Omentum	Positive	None	0.427
T9	Stage 3	Omentum	Positive	None	0.254
T10	Stage 3	Omentum	Positive	None	0.566
NACT0	Stage 4	Omentum	Positive	Carboplatin + Paclitaxel	0.620
NACT2	Stage 3	Omentum	Positive	Carboplatin + Paclitaxel	4.272
NACT3	Stage 3	Omentum	Positive	Carboplatin + Paclitaxel	1.145
NACT4	Stage 3	Omentum	Positive	Carboplatin + Paclitaxel	2.906
NACT5	Stage 3	Omentum	Positive	Carboplatin + Paclitaxel	1.336
NACT8	Stage 3	Omentum	Positive	Carboplatin + Paclitaxel	0.219
NACT9	Stage 4	Omentum	Positive	Carboplatin + Paclitaxel	1.134
NACT11	Stage 3	Omentum	Positive	Carboplatin + Paclitaxel	0.563
NACT12	Stage 3	Omentum	Positive	Carboplatin + Paclitaxel	4.075

Table S6 | Patient cohort characteristics for primary breast tumor tissues from triple-negative breast cancer patients treated with and without neoadjuvant platinum chemotherapy.

Patient Characteristics (Tumor Tissues)					
Triple Negative Breast Cancer					
**Patient ID**	**Clinical Staging**	**Tissue**	**Pathological Nodal Status**	**Neoadjuvant Chemotherapy**	**LVD (# LV/mm^2 tissue)**
NA1	Stage 1b	Mammary	pN0	None	0.12127603
NA2	Stage 1c	Mammary	pN0	None	0.25062657
NA3	Stage 1c	Mammary	pN0	None	0.22665947
NA4	Stage 1c	Mammary	pN0	None	0.66242999
NA5	Stage 2	Mammary	pN0	None	0.28330206
NA6	Stage 2	Mammary	pN0	None	0.39651071
NA8	Stage 2	Mammary	pN0	None	0.80311657
NA10	Stage 2	Mammary	pN0	None	0.19519879
NA11	Stage 1b	Mammary	pN0	None	0.18748535
NA13	Stage 1b	Mammary	pN0	None	0.4830745
NA14	Stage 1b	Mammary	pN0	None	0.26932668
NA16	Stage 1b	Mammary	pN0	None	0.30495553
NA18	Stage 1c	Mammary	pN0	None	0.45425667
NA20	Stage 2	Mammary	pN0	None	0.5240027
PA1	Stage 1c	Mammary	pN1	None	0.5138908
PA2	Stage 2	Mammary	pN1	None	0.32744592
PA3	Stage 1a	Mammary	pN1	None	0.12771678
PA4	Stage 1c	Mammary	pN1	None	0.2988741
PA5	Stage 3	Mammary	pN1a	None	0.22340023
PA7	Stage 2	Mammary	pN1mi	None	0.78431373
PA8	Stage 1a	Mammary	pN1a	None	0.4642765
NN4	Stage 2	Mammary	ypN0	Cisplatin + Paclitaxel	1.21918721
PN5	Stage 3a	Mammary	ypN3a	Cisplatin + Paclitaxel	0.29317125
PN6	Stage 1a	Mammary	ypN1	Cisplatin + Paclitaxel	0.2788409
NN5	Stage 1c	Mammary	ypN0	Cisplatin + Paclitaxel	0.49746661
PN3	Stage 2	Mammary	ypN1a	Cisplatin + Paclitaxel	1.51354924
PN11	Stage 1c	Mammary	ypN1	Carboplatin + Paclitaxel	0.93373494
